# Salivary Oxytocin Concentrations in Males following Intranasal Administration of Oxytocin: A Double-Blind, Cross-Over Study

**DOI:** 10.1371/journal.pone.0145104

**Published:** 2015-12-15

**Authors:** Katie Daughters, Antony S. R. Manstead, Kelly Hubble, Aled Rees, Anita Thapar, Stephanie H. M. van Goozen

**Affiliations:** 1 School of Psychology, Cardiff University, Cardiff, United Kingdom; 2 Institute of Molecular and Experimental Medicine, School of Medicine, Cardiff University, Cardiff, United Kingdom; 3 Institute of Psychological Medicinal and Clinical Neurosciences, School of Medicine, Cardiff University, Cardiff, United Kingdom; University of Tokyo, JAPAN

## Abstract

The use of intranasal oxytocin (OT) in research has become increasingly important over the past decade. Although researchers have acknowledged a need for further investigation of the physiological effects of intranasal administration, few studies have actually done so. In the present double-blind cross-over study we investigated the longevity of a single 24 IU dose of intranasal OT measured in saliva in 40 healthy adult males. Salivary OT concentrations were significantly higher in the OT condition, compared to placebo. This significant difference lasted until the end of testing, approximately 108 minutes after administration, and peaked at 30 minutes. Results showed significant individual differences in response to intranasal OT administration. To our knowledge this is the largest and first all-male within-subjects design study to demonstrate the impact of intranasal OT on salivary OT concentrations. The results are consistent with previous research in suggesting that salivary OT is a valid matrix for OT measurement. The results also suggest that the post-administration ‘wait-time’ prior to starting experimental tasks could be reduced to 30 minutes, from the 45 minutes typically used, thereby enabling testing during peak OT concentrations. Further research is needed to ascertain whether OT concentrations after intranasal administration follow similar patterns in females, and different age groups.

## Introduction

The use of intranasal oxytocin (IN-OT) in scientific research has become increasingly popular over the past decade. According to a recent review, 230 papers have reported using IN-OT since 1958 [1]. This scientific interest spans several fields, from clinical psychology, with respect to autism spectrum disorder [2, 3] and schizophrenia [4], to social psychology, with respect to intergroup relationships [5] and emotional processing [6]. Despite this flourishing interest, concern has been expressed that the assumptions upon which this line of research depends have not been securely established. In particular, there is a lack of evidence concerning both the longevity of the effects of intranasal spray on peripheral OT concentrations and the pattern of concentrations during these effects [1]. Few studies have addressed these questions, and many IN-OT administration studies do not include any assessment of participants’ OT concentrations. The aim of the present study was to provide evidence that IN-OT has a significant impact on salivary OT concentrations in healthy adults (which cannot be explained by ‘spiking’ alone; see below), and the nature of this impact. We used a double-blind, cross-over design.

We begin by addressing questions about the validity of saliva testing [7]. Although others [8–10] have addressed these concerns in detail, we note that there have been recent improvements in the preferred commercial saliva ELISA (enzyme linked immunosorbent assay) that is commonly used in OT research [11]. These improvements have sought to address the main concern raised by McCullough et al. [7], namely that earlier ELISAs had a high rate of non-specific binding (when non-OT compounds bind to ‘OT-specific’ antibodies), leading to artificially elevated concentrations of OT. The latest ELISA kit [11] has reduced non-specific binding, and thereby alleviates this problem. We used this latest kit.

How long IN-OT remains elevated in saliva remains unclear. According to Veening and Olivier [1], nearly 80 papers that reported using IN-OT administration were published in 2012. To our knowledge only three of these investigated the patterns of OT concentrations in saliva in healthy adults after IN-OT administration. One study [12] found that salivary OT was still elevated 7 hours after administration in a double-blind, between-subjects study (n = 46; all female). Participants in both the high dose (24 IU; n = 10) and low dose (16 IU; n = 18) IN-OT conditions still had significantly higher salivary OT concentrations after 7 hours, compared to participants in the placebo condition. Concentrations in both OT conditions ranged from tenfold to one hundredfold the average placebo concentration. However, there was no statistically significant difference between the high and low dose OT conditions at any point in the study.

There are reasons to question the generalizability of these findings concerning longevity, because there is no other evidence that IN-OT causes elevated OT concentrations for such an extended period of time. Weisman, Zagoory-Sharon, and Feldman [13] sampled salivary OT concentrations over a 4-hour period after IN-OT administration in 10 participants (5 female; within-subjects design). Samples were taken at baseline and 15, 30, 45, 60, 80, 100, 120, 180, 240 minutes after administration. OT concentrations were significantly higher 240 minutes after administration, compared to the placebo condition. There was a significant decrease in salivary OT between 30 minutes and 45 minutes post-administration, followed by a plateau phase lasting from 45 minutes to 120 minutes after administration. Due to the time intervals between samples, it is difficult to state exactly when this plateau ceased. It is possible that the plateau lasted for some time past 120 minutes before salivary OT decreased significantly at 180 minutes.

Although Weisman et al. [13] reported a significant increase in salivary OT at 15 minutes post-administration, the precise values should be treated with caution. Given the mode of delivery, is it possible that at 15 minutes what is actually being measured is Syntocinon (synthetic OT) spray that has trickled down from the nasal cavity to the back of the throat, causing an artificial ‘spike’ in OT concentrations in the saliva. This can occur if some of the OT spray is not absorbed across the nasal membrane during administration. In this instance, the fine hairs in the nasal cavity move the substance from the front of the nasal cavity, to the back, and down the throat. Here the synthetic OT spray could then be brought forward into the mouth when participants are asked to provide a saliva sample.

This process of clearing substances from the nasal cavity to the back of the throat, called mucociliary transport, takes 12–15 minutes in healthy individuals [14]. Hence, taking saliva samples at 15 minutes creates the risk of collecting saliva that is ‘spiked’ with IN-OT (indeed this may be why other saliva studies have chosen 30 minutes as their first measurement, as this allows almost double the length of time of average mucociliary transport [14]). Importantly, when substances are brought to the back of the throat, they do not remain there indefinitely; instead, the swallow reflex is activated and moves substances from the throat into the oesophagus and down to the stomach. Therefore IN-OT that is not absorbed across the nasal membrane is moved to the back of the throat, where it is then swallowed, and can therefore no longer ‘spike’ saliva. Because this process takes up to 15 minutes on average, spiking cannot account for the sustained and highly significant effects of IN-OT on salivary OT concentrations reported in the literature.

It is nonetheless worth noting that concentrations may peak earlier than 30 minutes, as is the case for many other small peptides [1], and can be reliably measured in other matrices. A recent study [15] investigating the relationship between IN-OT and its impact on OT concentrations in plasma and cerebrospinal fluid found that plasma OT was significantly elevated 15 minutes post-administration. A second study [16] also found significantly elevated plasma OT concentrations at 10 minutes post-administration.

One of the largest studies to empirically test the longevity of IN-OT in saliva was conducted by Huffmeijer et al. [17]. Fifty-seven females took part in the study, which had a between-subjects design. Salivary OT concentrations were still significantly elevated 2.25 hours after administration, compared to placebo concentrations. However, only three samples were taken during the study (baseline, 1.25 and 2.25 hours after administration) so few conclusions can be drawn regarding the pattern of OT concentrations during this elevation, except to corroborate other evidence that the impact of IN-OT lasts for at least 90 minutes in both plasma and saliva [18].

### The Present Study

We predicted that IN-OT would lead to a significant increase in salivary OT concentrations, compared to placebo, in healthy adult males. With just a handful of studies having found significantly elevated concentrations at approximately 120 minutes, we set out to establish both the pattern of the intranasal administration effects on salivary OT, and to confirm that these effects can last up to and beyond 90 minutes.

To achieve this aim we administered either a single dose of 24 IU of IN-OT or a placebo to male participants in a double-blind, cross-over design. Saliva samples were collected at baseline, and then at 30, 60, 90, 105, and 108 minutes after administration. Participants completed a series of psychological tasks during the post-baseline period. These tasks were matched in the OT and placebo conditions with respect to timing and content. Performance on these tasks is not the central concern of the present paper, which focuses solely on salivary OT concentrations.

## Materials and Methods

### Participants

Forty healthy male students (M age = 20.98; SD = 4.55) from Cardiff University took part in the study. The majority were Psychology students (n = 31); those studying other disciplines were Chemistry, Engineering or Journalism students. Psychology students were awarded course credits; non-Psychology students received financial compensation for their time.

### Procedure

Each of the two testing sessions lasted approximately 3 hours. There was a 2-week interval between the two sessions (for practical reasons seven participants had to be tested at later dates; the longest interval between the two sessions was 35 days). The two sessions were timed to take place at the same time of day to control for any potential diurnal effects. Participants were instructed to abstain from alcohol for 24 hours, and caffeine for 2 hours prior to testing. All participants were non-smokers. Participants were only allowed to drink water during the sessions; if any food had been consumed before the start of a session, participants were asked to rinse their mouths thoroughly before any saliva samples were taken.

On arrival participants completed a series of questionnaires and provided their baseline saliva sample 30 minutes after arrival, to allow for acclimatisation to the testing facility. Participants then self-administered 24 IU (three puffs per nostril, at their own pace) of synthetic OT or an independently manufactured placebo nasal spray that chemically matched the OT spray for all compounds, except OT. Both sprays were manufactured by St Mary’s Pharmaceutical Unit, Cardiff (http://www.wales.nhs.uk/sites3/home.cfm?orgid=828). Recommendations regarding administration procedures made by Guastella et al. [19] were followed, although participants’ nasal cavities were not medically examined. A doctor was present during administration and for the next 15 minutes. Half an hour after administration participants provided their second saliva sample, after which they began completing the experimental tasks.

To assess whether the effect of the OT spray remained detectable in saliva for up to 90 minutes (based on previous literature), two further samples were taken at 30 min intervals: 60 min and 90 min post-administration. The final two saliva samples were taken at approximately 105 and 108 minutes after administration, immediately before and immediately after a video excerpt that was intended to evoke an empathic response. The 108-minute saliva sample marked the end of the testing session.

The study was approved by both the School of Psychology Ethics Committee at Cardiff University, and the Research and Development Office at Cardiff and Vale University Health Board. All participants completed medical pre-screening forms and signed statements of health before leaving each testing session. They were also cleared to participate in the study by a medical professional. Participants gave written informed consent at the start of both testing sessions, and were fully debriefed after their second session.

### Oxytocin Sampling and Analysis

Saliva samples were collected in pre-chilled polypropylene 5ml tubes (Sarstedt, Leicester, UK) that were stored on ice throughout the session (the type of plastic used for saliva collection is important as different proteins bind more strongly to certain types of plastic, which can result in inaccurate sampling [20]). For each sample, participants were asked to produce 2ml of passive drool. Samples were frozen as quickly as possible during testing, and were left on ice for no longer than 1 hour. Samples were frozen at -80°C to ensure that they remained stable during long-term storage (the first samples collected were stored for 6 months; the final samples were frozen for a day). Once all samples had been collected, they were thawed and centrifuged at 4°C at 1600 x g for 15 min; 1ml of supernatant was transferred to a new tube before being frozen again at -80°C. To ensure placebo and baseline saliva samples would be above the minimum sensitivity of the ELISA (15pg/ml) the samples had to be concentrated. Although the kit manual provides instructions for a chemical concentration process, it is also possible to lyophilize (freeze dry) samples, effectively achieving the same outcome [8, 10]. Lyophilization has also been found to significantly increase the validity of measuring OT via ELISA [7, 21, 22]. Therefore, we used the lyophilisation process instead of the chemical process outlined in the manual. Samples were freeze-dried overnight (for approximately 15 hours), until all samples were dehydrated. The length of freeze-drying required depends upon the volume added. Because samples were freeze-dried in batches due to a limited space, some batches contained more total volume than others, therefore requiring a slightly longer time to achieve the same outcome, compared to other batches. After samples were lyophilized they were stored at -20°C until analysis. It was appropriate to store freeze-dried samples at -20°C for two reasons: first, samples become more stable when they have been freeze-dried; second, samples were to be analyzed within 2 weeks of freeze-drying, and therefore only required short-term storage.

Samples were analyzed using a 96-well OT ELISA kit (Enzo Life Sciences, Exeter, UK). This kit has been used in several OT studies [12, 13] although as noted above the kit has recently (September 2013) undergone modification and “rigorous validation” [[11] p, 1]. As a result, the ELISA used in the present study has a greater specificity compared to the earlier version. Freeze dried samples were reconstituted in 250μl of assay buffer, thereby concentrating all samples four-fold. Where possible all samples were run in duplicate. Some participants struggled to produce enough saliva for every sample, in which case only 0.5 ml was frozen after centrifugation. In such cases, samples were then reconstituted in 125μl of assay buffer, such that the concentration was the same as the other samples; however in these cases there was only sufficient volume to run a single analysis. All samples were processed in accordance with the manual’s ELISA protocol [23], with an overnight incubation of 19 hours. Samples were read at 405nm and concentrations were calculated from the standard curve. Finally, the international correction for OT concentrations, devised by the National Institute for Biological Standards and Control and the World Health Organisation was applied.

The ELISA manual [23] reports that intra-assay and inter-assay coefficients of variability are 12.6–13.3% and 11.9–20.9%, respectively. The present study obtained intra-assay and inter-assay coefficients of <8% and 10.6–14.5%, respectively. Accepted values for coefficients of variability are <10% for intra-assay and <15% for inter-assay variability [24]. To confirm that the process of freeze drying did not significantly degrade samples, a serial dilution series was prepared and freeze dried with the samples. There was a high correlation between the control series and the standards (*r*(6) = .96, *p* = 0.002).

Because a number of samples were more than three standard deviations above the mean, the data were winsorized prior to data analysis.

## Results

Initial analysis revealed that 9 out of 12 samples violated the assumption of normality (see S1 Table). We therefore used non-parametric tests to assess whether there was a significant effect of drug on salivary OT concentrations.

Mean salivary OT concentrations are shown in Table 1, broken down by Drug (OT and placebo) and Time (Samples 1–6). Related-samples Friedman’s two-way analysis of variance revealed a significant difference between samples, χ^2^(11) = 286.75, p <0.001. Follow-up Wilcoxon signed rank tests were then carried out. The results of these tests are also shown in Table 1. There was no significant difference between the baseline samples. There were significant differences between all the remaining samples.

**Table 1:** Mean salivary oxytocin concentrations (pg/ml) at each time point for OT and placebo condition, with reported outcomes of Wilcoxon signed rank test.

Time	OT Condition	Placebo Condition	*Z*	Sig
	Mean(SD)	Mean(SD)		
**Baseline**	77.93(74.46)	45.96(33.27)	-1.91	.056
**30 minutes**	999.52(813.95)	37.45(21.24)	-5.43	<.001
**60 minutes**	951.98(772.96)	46.43(33.54)	-5.50	<.001
**90 minutes**	531.29(538.76)	39.58(29.77)	-5.38	<.001
**105 minutes**	417.41(366.88)	44.88(33.47)	-5.31	<.001
**108 minutes**	355.17(235.69)	34.08(25.97)	-5.50	<.001

**Fig 1:**
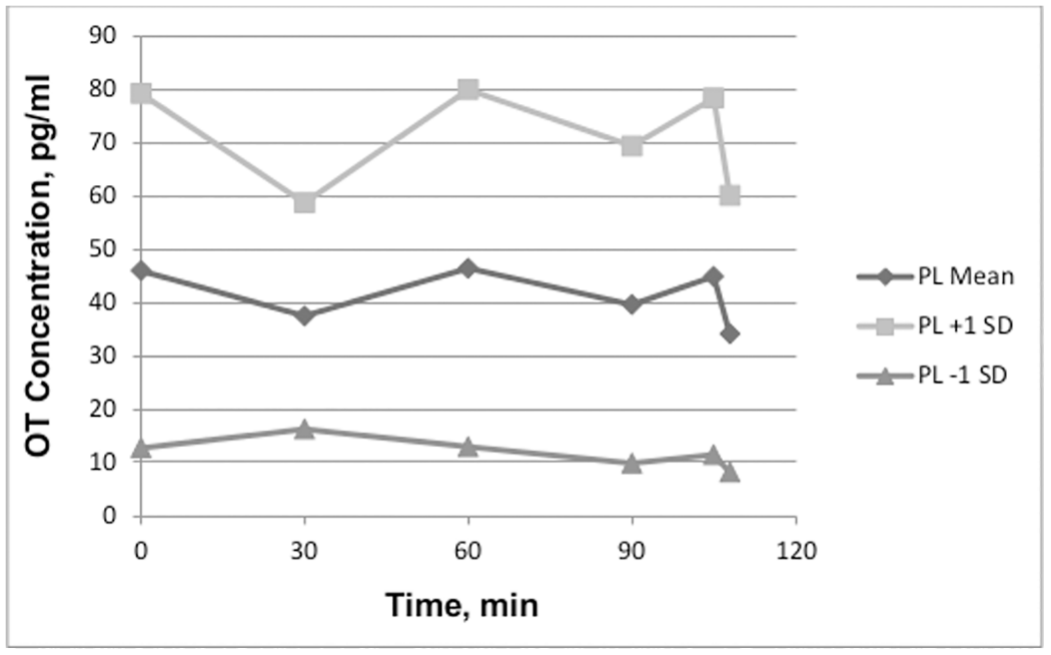
The mean and +/- 1 standard deviation of OT concentrations in the placebo condition.

**Fig 2:**
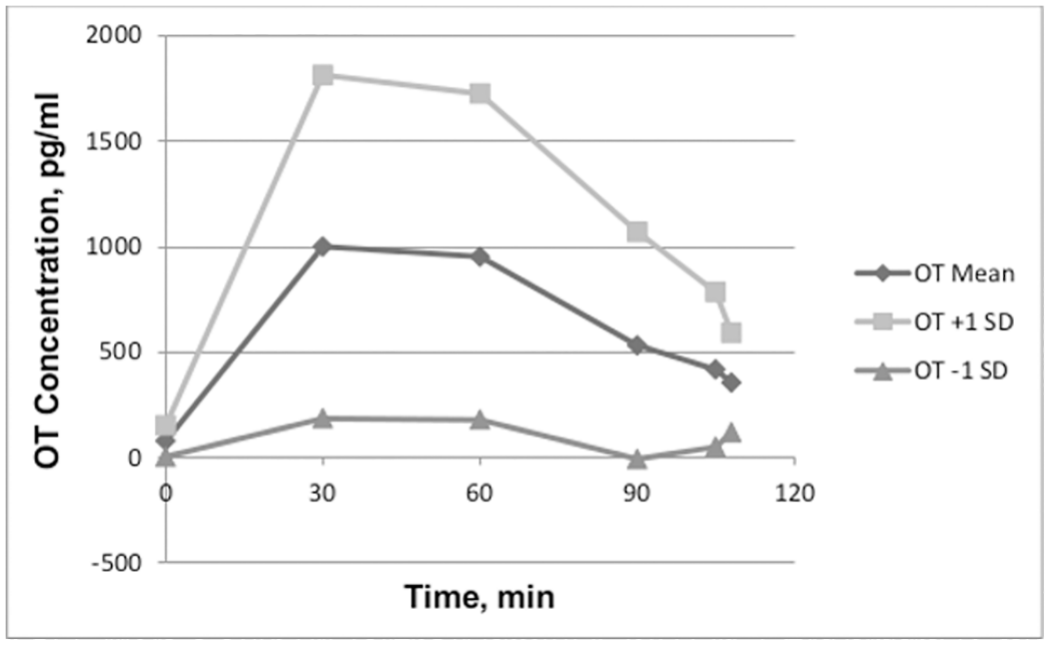
The mean and +/- 1 standard deviation of OT concentrations in the oxytocin condition.

Large standard deviations showed that there were considerable individual differences in OT concentrations after In-OT. To demonstrate this, the means for each drug condition are presented in Figs 1 and 2, with additional lines representing +/- 1 standard deviation. All participants’ OT concentrations for both conditions are presented in S2 Table.

## Discussion

To our knowledge, this is the largest within-subjects design study to date demonstrating that intranasal administration of OT has significant effects on salivary OT concentrations in males. As expected, participants had significantly higher salivary OT concentrations after OT administration, compared to their placebo session. In line with previous research [18, 25–27], maximum OT concentrations were detected 30 minutes after administration. When using saliva sampling, 30 minutes may be the first reliable time point at which to measure OT concentrations, given that samples taken prior to this may reflect ‘spiking’. Samples taken from 30 minutes onwards, however, cannot reflect spiking, as substances that are not absorbed across the nasal membrane are transported to the back of the throat, and then swallowed within 15 minutes [14]. Any remaining IN-OT at 30 minutes could not account for the significant increase seen here, and also in other studies.

The results are also consistent with previous findings [13, 17] in showing that the effect of IN-OT on peripheral (salivary in the present study) OT concentrations lasts beyond 90 minutes. Together, these results support the idea of a positive feedback system between peripheral and central concentrations of OT: “… central regulating mechanisms [control] the duration of the elevated levels of peripheral OT” [[1] p. 1450]. This would explain why a compound with a half-life of several minutes in peripheral bodily fluids remains elevated for such an extended period of time. Although the mechanism by which IN-OT affects both central and peripheral concentrations both directly and indirectly is not fully understood [an issue raised in several papers [22, 28]], the continuing interest in OT research has driven biologists as well as psychologists and clinicians to investigate this further [e.g., [29]]. A pioneering study [26] showed that IN-OT does reach specific brain regions in rats and mice, demonstrating that IN-OT does cross the blood-brain barrier, thereby addressing one of the main concerns [28]. More recently, Paloyelis et al. [27] demonstrated that IN-OT also crosses the blood-brain barrier in human males, providing further evidence that IN-OT has direct effects on the brain. In addition, several studies have found that IN-OT leads to a significant increase in OT concentrations in cerebrospinal fluid, which may also be indicative of IN-OT reaching the brain [30, 31]. However, more research is required to corroborate these findings, and evaluate the relationship between central and peripheral concentrations of OT.

An important attribute of the present study is its use of a within-subjects design. The extent of individual differences in response to IN-OT is considerable and thus far unaddressed in the literature. Indeed, this is an important area of focus for research, especially psychological research, partly because these individual differences may be shaped by psychological factors, and partly because individual differences in reactivity to IN-OT are likely to influence subsequent psychological and behavioral variables. In addition to Guastella et al.’s [19] suggestion that anatomical differences could account for individual differences in response to IN-OT, and the possibility that genetic factors may also influence responses, individual differences in psychological factors might account for a significant proportion of this variance. Several studies have shown that individual differences in social anxiety and early parental relationships can moderate the way in which participants respond behaviorally to IN-OT [5, 32, 33]. Investigating or controlling for these variables in future studies may advance our understanding of their effect on OT-mediated processes.

Furthermore, a recent paper expressed concern regarding the statistical power of many IN-OT studies [34], and we have already expressed our own concern that few studies reporting behavioural effects of IN-OT include a measure of peripheral OT. By using a within-subjects design, and the largest, within-subjects, sample size published to date, we aimed to address both types of concerns.

Some limitations of the present research should be acknowledged. First, although the baseline concentrations are statistically similar, the difference does not fall very far short of statistical significance, with OT baseline concentrations being higher than placebo baseline concentrations. We have carefully reviewed possible reasons for this (non-significant) difference. The study was double-blind; participants were randomly assigned to their drug order; there were no significant order effects; and all data points were winsorized (therefore there were no statistical outliers). Within the winsorized dataset, there were four participants with noticeably higher OT baseline concentrations; if their values are removed the OT baseline mean decreases to 51.13 (SD = 30.86) pg/ml, much closer to the placebo baseline mean of 45.96 pg/ml. However, in absence of any good reason to remove them, we retained their winsorized values in the dataset. These atypical values may results from individual differences that are either psychological or anatomical/genetic in origin. It is worth emphasizing that this small and non-significant difference in baseline values cannot account for the much larger and highly significant difference in salivary OT concentrations following intranasal administration, and therefore does not detract from the main result of the present study.

One other possible limitation is that the concentrations of OT observed in the placebo condition are higher than those previously reported. Typical values observed in previous research suggest that OT concentrations in peripheral bodily fluid are <10 pg/ml under placebo conditions [e.g., [35]]. In the present study we found concentrations of 30–40 pg/ml across the testing session. It is important to note that although many studies use the same commercial ELISA kit, there is no standard operating procedure for saliva collection (an issue raised by [19]). Given that salivary OT can degrade very quickly and the lack of a standardised procedure for its collection, it is difficult to compare concentrations between studies. There are three reasons why these apparently elevated placebo concentrations may not be of real concern: 1) placebo concentrations were highly consistent within participants; 2) there is no methodological reason to question the validity of the concentrations; 3) the primary effects found in the present study are the same as those observed in other studies.

A final limitation of the present study concerns the number of saliva samples taken. Due to logistical and financial restrictions, it was not possible to collect samples beyond 108 minutes. As previously stated, the longevity of IN-OT effects on peripheral bodily fluids is not established. Given the interest in IN-OT studies, and its known effects on behavior, it is important to address this question in future studies.

In light of the present findings, taken together with those from previous research, it is recommended that the ‘wait-time’ between intranasal administration of OT and experimental testing should be no longer than 30 minutes. Several studies have now shown that OT concentrations in both plasma [18, 26] and saliva [13] peak at 30 minutes, if not earlier. Testing should therefore begin at 30 minutes to ensure that tasks are carried out under peak OT concentrations. This also aids the efficiency of testing. If testing begins 30 minutes post administration then, based on current research, one further hour of testing can be conducted whilst endogenous OT concentrations are significantly elevated above baseline.

As previously noted, however, there is a need to develop standard operating procedures for all aspects of OT research: sampling, processing of samples, intranasal administration techniques, and how OT studies are reported. This would enable precise replications and more accurate comparisons between studies to be made.

In terms of saliva analysis, it would be beneficial if future research could establish whether freeze drying samples rather than using chemical extraction results in higher concentrations. In addition, shorter time intervals between samples (for example, as used by [13]) would enable a more detailed picture of the pattern of OT concentrations after IN-OT administration.

Finally, future research could investigate whether different age groups and whether males and females, differ in responsiveness to IN-OT administration. The present study and the cited literature provide evidence of the effects of IN-OT in young adults. However, we are unable to assess whether this is representative of responsiveness in older adults or in children. Although one research group has investigated peripheral OT concentrations in response to OT-related behavior across two generations [36], those researchers analyzed ‘natural’ OT concentrations before and after social interaction. To our knowledge, no studies have examined whether there are differences in responsiveness to intranasal administration of OT across generations. Given the interest in the therapeutic potential of IN-OT (e.g., [1]), this is a high priority research question.

In conclusion, the present study found that intranasal administration of OT resulted in a significant increase in salivary OT concentrations in healthy adult males for at least 108 minutes. The findings underscore the need for a within-subjects design when employing a placebo controlled, IN-OT study, because of the large individual differences both in baseline concentrations and especially in response to OT sprays. It would be desirable for future researchers to include a manipulation check for endogenous OT. This would not only add to the relatively small literature on the effects of IN-OT on peripheral OT concentrations, but also enable researchers to identify individuals who are more responsive, compared to others, and to assess whether there are any psychological or behavioral differences between more and less responsive individuals.

## Supporting Information

S1 Table Results of Shapiro-Wilks tests for normality for each saliva sample. (DOCX)

S2 Table Salivary OT concentrations for Placebo and Oxytocin conditions. (DOCX)
