# Supplementary material for: Salivary Oxytocin Concentrations in Males following Intranasal Administration of Oxytocin: A Double-Blind, Cross-Over Study
Source: PLoS One. 2015 Dec 15;10(12):e0145104. doi: 10.1371/journal.pone.0145104 (PMC4684402; doi:10.1371/journal.pone.0145104)
Supplement: S1 Table — (DOCX) [file pone.0145104.s001.docx]

**S1 Table. Results of Shapiro-Wilks tests for normality for each saliva sample**

| **Saliva sample** | ***D*** | **df** | ***p*** |
| --- | --- | --- | --- |
| Placebo Baseline | .150 | 39 | .027 |
| Placebo 30 mins | .110 | 39 | >.05 |
| Placebo 60 mins | .160 | 39 | .013 |
| Placebo 90 mins | .139 | 39 | >.05 |
| Placebo 105 mins | .221 | 39 | <.001 |
| Placebo 108 mins | .200 | 39 | <.001 |
| Oxytocin Baseline | .239 | 39 | <.001 |
| Oxytocin 30 mins | .186 | 39 | .002 |
| Oxytocin 60 mins | .179 | 39 | .003 |
| Oxytocin 90 mins | .200 | 39 | <.001 |
| Oxytocin 105 mins | .139 | 39 | >.05 |
| Oxytocin 108 mins | .170 | 39 | .006 |
