# Supplementary material for: Salivary Oxytocin Concentrations in Males following Intranasal Administration of Oxytocin: A Double-Blind, Cross-Over Study
Source: PLoS One. 2015 Dec 15;10(12):e0145104. doi: 10.1371/journal.pone.0145104 (PMC4684402; doi:10.1371/journal.pone.0145104)
Supplement: S2 Table — (DOCX) [file pone.0145104.s002.docx]

**S2 Table***.* **Salivary OT concentrations for placebo and oxytocin conditions**

| Participant | Session | Condition | Baseline | 30 | 60 | 90 | 105 | 108 |
| --- | --- | --- | --- | --- | --- | --- | --- | --- |
| 1 | 1 | Placebo | 31.25 | 28.22 | 16.44 | 31.25 | 51.14 | 32.60 |
|  | 2 | Oxytocin | 34.82 | 538.60 | 701.51 | 148.96 | 232.32 | 288.40 |
| 2 | 1 | Placebo | 29.08 | 8.85 | 29.26 | 45.90 | 40.95 | 21.80 |
|  | 2 | Oxytocin | 269.96 | 1959.14 | 1947.40 | 281.55 | 987.89 | 221.43 |
| 3 | 1 | Placebo | 36.32 | 22.60 | 5.61 | 7.44 | 17.97 | 14.02 |
|  | 2 | Oxytocin | 15.95 | 1801.14 | 845.07 | 380.17 | 84.70 | 206.03 |
| 4 | 1 | Placebo | 36.21 | 28.56 | 157.55 | 19.95 | 35.81 | 30.52 |
|  | 2 | Oxytocin | 33.39 | 624.10 | 241.60 | 213.99 | 157.11 | 103.87 |
| 5 | 1 | Placebo | 14.57 | 39.12 | 42.49 | 38.69 | 32.78 | 43.44 |
|  | 2 | Oxytocin | 27.94 | 799.99 | 421.82 | 177.38 | 289.85 | 288.26 |
| 6 | 1 | Oxytocin | 232.45 | 761.24 | 233.74 | 115.35 | 565.10 | 831.50 |
|  | 2 | Placebo | 32.78 | 33.15 | 19.09 | 18.47 | 31.89 | 31.20 |
| 7 | 1 | Placebo | 62.23 | 41.44 | 82.02 | 71.99 | 71.99 | 36.09 |
|  | 2 | Oxytocin | 68.23 | 3043.75 | 2846.97 | 1719.60 | 1357.65 | 1137.94 |
| 8 | 1 | Placebo | 17.96 | 38.08 | 17.15 | 37.22 | 37.22 | 18.52 |
|  | 2 | Oxytocin | 63.68 | 1423.53 | 2846.97 | 558.32 | 558.32 | 357.80 |
| 9 | 1 | Oxytocin | 78.54 | 2670.42 | 1979.87 | 753.05 | 753.05 | 161.11 |
|  | 2 | Placebo | 1.88 | 23.13 | 24.03 | 21.26 | 21.26 | 14.94 |
| 10 | 1 | Oxytocin | 19.39 | 635.01 | 340.22 | 102.82 | 65.96 | 44.26 |
|  | 2 | Placebo | 41.23 | 27.50 | 15.92 | 19.43 | 16.44 | 18.81 |
| 11 | 1 | Oxytocin | 301.31 | 1383.11 | 1133.03 | 1231.86 | 561.94 | 389.43 |
|  | 2 | Placebo | 135.71 | 67.25 | 100.85 | 115.54 | 47.51 | 34.89 |
| 12 | 1 | Oxytocin | 14.83 |  | 128.78 | 1719.60 | 875.95 | 223.95 |
|  | 2 | Placebo | 20.19 | 22.38 | 27.15 | 29.90 | 40.98 | 20.85 |
| 13 | 1 | Placebo | 11.46 | 10.68 | 7.08 | 17.19 | 17.30 | 8.39 |
|  | 2 | Oxytocin | 61.91 | 106.34 | 44.18 | 16.58 | 79.35 | 25.24 |
| 14 | 1 | Placebo | 18.01 | 18.24 | 15.56 | 30.16 | 12.53 | 11.25 |
|  | 2 | Oxytocin | 63.91 | 549.27 | 78.85 | 21.94 | 19.07 | 22.36 |
| 15 | 1 | Placebo | 15.56 | 22.51 | 24.92 | 16.06 | 24.60 | 15.56 |
|  | 2 | Oxytocin | 147.11 | 186.17 | 431.28 | 94.83 | 124.68 | 81.92 |
| 16 | 1 | Placebo | 59.26 | 56.93 | 71.81 | 41.67 | 36.08 | 36.08 |
|  | 2 | Oxytocin | 99.70 | 209.86 | 344.70 | 260.48 | 75.94 | 33.84 |
| 17 | 1 | Oxytocin | 88.42 | 1201.37 | 1260.47 | 1040.19 | 456.14 | 872.25 |
|  | 2 | Placebo | 19.02 | 20.11 | 26.62 | 68.99 | 93.51 | 16.73 |
| 18 | 1 | Placebo | 19.02 | 36.95 | 28.38 | 23.99 | 55.58 | 20.60 |
|  | 2 | Oxytocin | 56.03 | 1343.82 | 2053.82 | 796.25 | 650.25 | 511.46 |
| 19 | 1 | Oxytocin | 21.09 | 64.98 | 71.10 | 21.57 | 38.72 | 49.23 |
|  | 2 | Placebo | 20.62 | 29.78 | 42.69 | 15.51 | 50.73 | 31.15 |
| 20 | 1 | Placebo | 16.10 | 7.78 | 12.76 | 9.96 | 12.85 | 7.11 |
|  | 2 | Oxytocin | 105.82 | 527.04 | 675.10 | 119.32 | 151.70 | 147.21 |
| 21 | 1 | Oxytocin | 25.63 | 32.83 | 96.71 | 89.72 | 72.18 | 1137.94 |
|  | 2 | Placebo | 35.39 | 62.12 | 98.17 | 138.64 | 190.54 | 62.44 |
| 22 | 1 | Oxytocin | 301.31 | 245.75 | 688.99 | 548.32 | 43.61 | 217.10 |
|  | 2 | Placebo | 47.47 | 37.78 | 23.16 | 36.33 | 28.54 | 140.45 |
| 23 | 1 | Placebo | 53.74 | 91.16 | 49.05 | 41.66 | 44.18 | 52.01 |
|  | 2 | Oxytocin | 57.36 | 1239.48 | 716.50 | 373.12 | 573.95 | 264.04 |
| 24 | 1 | Oxytocin | 34.93 | 1673.35 | 1619.64 | 31.68 | 32.94 | 17.72 |
|  | 2 | Placebo | 19.55 | 24.08 | 19.29 | 21.14 | 10.25 | 13.61 |
| 25 | 1 | Placebo | 53.84 | 50.76 | 60.18 | 70.42 | 48.80 | 57.48 |
|  | 2 | Oxytocin | 30.66 | 1789.17 | 794.32 | 1719.60 | 383.98 | 611.27 |
| 26 | 1 | Placebo | 71.82 | 50.76 | 34.04 | 69.96 | 47.23 | 54.19 |
|  | 2 | Oxytocin | 100.96 | 2340.22 | 2177.57 | 1161.31 | 1357.65 | 979.50 |
| 27 | 1 | Placebo | 84.27 | 15.07 | 84.83 | 18.39 | 76.28 | 18.15 |
|  | 2 | Oxytocin | 74.27 | 351.87 | 435.27 | 102.19 | 149.27 | 24.64 |
| 28 | 1 | Placebo | 46.95 | 10.18 | 47.58 | 8.39 | 33.45 | 9.85 |
|  | 2 | Oxytocin | 24.97 | 273.32 | 726.20 | 113.66 | 459.05 | 49.19 |
| 29 | 1 | Oxytocin | 40.83 | 631.59 | 1644.95 | 335.87 | 1303.50 | 340.37 |
|  | 2 | Placebo | 52.57 | 13.82 | 27.95 | 6.35 | 23.05 | 16.43 |
| 30 | 1 | Placebo | 11.27 | 20.12 | 24.56 | 22.59 | 21.18 | 30.77 |
|  | 2 | Oxytocin | 87.35 | 810.94 | 2103.43 | 1719.60 | 282.03 | 376.84 |
| 31 | 1 | Oxytocin | 82.96 | 1885.30 | 639.00 | 731.54 | 469.08 | 93.16 |
|  | 2 | Placebo | 39.81 | 69.72 | 98.08 | 31.17 | 40.07 | 26.54 |
| 32 | 1 | Oxytocin | 6.39 | 1402.10 | 1264.66 | 1042.48 | 614.78 | 859.33 |
|  | 2 | Placebo | 18.98 | 17.92 | 20.78 | 8.65 | 17.13 | 17.13 |
| 33 | 1 | Oxytocin | 48.68 | 234.14 | 193.47 | 58.54 | 188.62 | 80.45 |
|  | 2 | Placebo | 38.48 | 60.82 | 37.27 | 32.61 | 47.76 | 32.20 |
| 34 | 1 | Placebo | 135.71 | 57.80 | 73.60 | 61.60 | 48.99 | 40.74 |
|  | 2 | Oxytocin | 45.97 | 185.05 | 439.40 | 508.60 | 486.46 | 349.50 |
| 35 | 1 | Oxytocin | 34.10 | 2086.58 | 1518.30 | 1719.60 | 668.53 | 668.53 |
|  | 2 | Placebo | 53.55 | 36.80 | 75.50 | 49.62 | 37.51 | 49.31 |
| 36 | 1 | Placebo | 35.28 | 32.37 | 40.80 | 45.66 | 41.08 | 36.95 |
|  | 2 | Oxytocin | 33.02 | 241.88 | 1122.51 | 1191.38 | 769.87 | 394.66 |
| 37 | 1 | Placebo | 105.79 | 70.20 | 72.56 | 50.76 | 57.18 | 34.36 |
|  | 2 | Oxytocin | 61.90 | 362.14 | 338.95 | 265.36 | 317.25 | 151.22 |
| 38 | 1 | Oxytocin | 67.46 | 2339.48 | 1199.29 | 490.95 | 545.77 | 687.98 |
|  | 2 | Placebo | 101.67 | 80.13 | 93.30 | 68.36 | 104.40 | 95.17 |
| 39 | 1 | Placebo | 92.77 | 42.85 | 33.37 | 20.61 | 21.38 | 20.61 |
|  | 2 | Oxytocin | 43.64 | 589.85 | 589.85 | 271.89 | 243.63 | 226.44 |
| 40 | 1 | Oxytocin | 46.67 | 437.49 | 320.55 | 190.89 | 106.95 | 548.23 |
|  | 2 | Placebo | 75.09 | 55.36 | 56.38 | 90.17 | 103.11 | 76.95 |
